# Global Access to Technology-Enhanced Medical Education During the COVID-19 Pandemic: The Role of Students in Narrowing the Gap

**DOI:** 10.9745/GHSP-D-20-00455

**Published:** 2021-03-31

**Authors:** Aleksander Dawidziuk, Michal Kawka, Bartosz Szyszka, Ignatius Wadunde, Aastha Ghimire

**Affiliations:** a Department of Medicine, Imperial College London, London, United Kingdom.; b School of Medicine, College of Health Sciences, Makerere University, Kampala, Uganda.; c Patan Academy of Health Sciences, Lagankhel Lalitpur, Nepal.

## Abstract

Althoughsome medical education institutions in high-income countries have the capacity to shift education to eLearning during the COVID-19 pandemic, educational institutions in low- and middle-income countries might struggle to fully implement it. We argue for medical students to advocate for national and international collaboration in adopting technology-enhanced learning globally.

## Key Messages

- The COVID-19 pandemic has severely affected medical education worldwide. With clinical placements suspended and social distancing rules in place, medical education institutions transitioned to online education to compensate for canceled in-person teaching.
- Although some institutions in high-income countries had the residual capacity to shift their education model to eLearning, educational institutions in low- and middle-income countries may have struggled to fully implement novel solutions due to obstacles such as slow and unstable bandwidth, limited access to computer facilities, and insufficient funding to develop online platforms.
- Some solutions have been postulated, but this issue has yet to be addressed. We argue that medical students have the responsibility to advocate for national and international collaboration to accelerate the adoption of technology-enhanced learning and hybrid teaching models globally.

## IMPACT OF COVID-19 PANDEMIC ON MEDICAL EDUCATION

The coronavirus disease (COVID-19) pandemic caused global disruption across all industries, resulting in nearly 72 million cases worldwide and more than 1.6 million deaths as of this writing.^1^ Due to the rapid spread of the illness in teaching hospitals and shortages in personal protective equipment, medical education was severely impacted. Clinical rotations were suspended, electives were canceled, and pre-clinical teaching had to be delivered remotely. In a survey distributed among final year medical students in the United Kingdom, more than 70% of respondents reported having their clinical placements canceled, and almost 50% had their final exams conducted in an altered format.^2^ Moreover, both international and national electives were called off, and pre-clinical teaching moved online.^3^ The General Medical Council, British Medical Association, and Medical Schools Council have expressed their concerns over the disruptions that the COVID-19 pandemic has caused for medical students in progressing through their studies and qualifying as doctors.

The disruptive effect of COVID-19 has been particularly severe for senior medical students whose teaching takes place mostly in clinical settings. The COVID-19 pandemic created a demand for digital resources to compensate for in-person teaching that was canceled due to social distancing requirements and the unavailability of clinical faculty.^4^ However, the technological transformation of medical education requires significant human and financial resources, which can be difficult to aggregate and mobilize when faced with a challenge like the COVID-19 pandemic.

Despite the limitations caused by the COVID-19 pandemic, a vast array of technology-enhanced learning (TEL) solutions was successfully introduced in high-income countries (HICs) in a very short time frame. Webinars conducted via Zoom, Skype, Google Hangouts, or WebX; online learning platforms; mobile applications; 3-dimensional anatomy models; and online question banks were implemented within the pre-existing infrastructure, thus facilitating their adoption. Examinations were altered to comply with the social distancing guidelines and were carried out as online tests, oral tests via teleconferences, and assessments of simulated tasks using video recordings. Moreover, novel approaches were developed to compensate for the lack of direct patient contact, including online video libraries of patient encounters and virtual consultation rooms.^6^

It is important to note that the majority of research on the implementation of TEL during the COVID-19 pandemic has been based on HIC experiences, and as such, solutions described might not be applicable in low-resource settings.^7^ An analysis of articles about medical education that were published during the first wave of the pandemic has revealed that of 28 articles that discussed TEL, only 2 had a first author based in a low- and middle-income country (LMIC). Thus, the overall positive conclusions of the HIC-focused articles might not be applicable to other settings. However, it is worth remembering that TEL is not devoid of limitations and poses challenges to both students and faculty in any setting. Although implementing TEL proves demanding in HICs, such a rapid adjustment can be extremely difficult in LMICs.^5^

Some institutions in HICs had the residual capacity to effectively shift to an online education model on short notice, but institutions in LMICs might struggle to implement these solutions. Infrastructural barriers including unstable bandwidth that results in low quality of audio and video outputs and slow download speed, as well as frequent electrical power failures, limit reliable access to TEL in real-time. Limited computer facilities and financial resources for the development of eLearning platforms and insufficient time available from the teachers to create digital content further hinder the transfer to remote education. Moreover, the successful delivery of teaching through online platforms depends on having technical support personnel available for teachers and students, which is also limited in LMICs.^8^

Also, because of the significant differences among LMICs and medical schools based there, there are unique challenges to be addressed by each university and its student community. In some institutions, before the COVID-19 pandemic, there were pre-existing online learning environments for students to use. However, many of these had limited interactivity, only allowed for administrative purposes like communicating test results, and were not regularly used for sharing educational resources. At the same time, many medical schools only relied on face-to-face and email communication. Considering the current medical crisis, the already present differences between these institutions were aggregated, as the development of pre-existing platforms was boosted by the needs created by the pandemic. At the same time, it was very difficult to build up the infrastructure without foundations.

We were able to witness some of these differences in capability in making technological adaptations between institutions in HICs and LMICs firsthand while undertaking clinical research electives in China and Thailand. We identified potential obstacles to the transfer of TEL solutions from HICs including restricted Internet access to certain search engines and social media portals that could be used for disseminating resources and incompatibility of computer systems with some eLearning platforms. Also, the clinical teachers were not accustomed to using digital assistance; instead, they relied on pen and paper. Additional technological training time may be required to prepare medical school faculty for TEL content delivery, making the process even more difficult.

We witnessed firsthand some of the differences in capability in making technological adaptations between medical schools in HICs and LMICs.

Despite the aforementioned challenges, due to the COVID-19 pandemic, institutions based in LMICs were forced to transition to TEL with mixed results. Intermittent Internet connectivity and lack of infrastructure for students to reliably access online teaching rendered the initial transition challenging both for students and educators.^9^^,^^10^ Maintaining stable Internet connections, which is essential for effective access to real-time remote teaching for students staying at home, is achievable, but due to the high cost of mobile data packages, it is not affordable for everyone. Further, although pre-clinical medicine courses are well-suited to be taught through webinars, clinical medicine, which relies on hands-on patient examination and acquiring practical skills, may require more advanced TEL solutions (e.g., virtual or mixed reality) to deliver high-quality education, most of which are not currently accessible in LMICs.^11^ Similar challenges also apply to the assessment of student competencies, which are an integral part of medical education.^12^

However, this does not mean that supplementing clinical education with online learning in LMICs should not be pursued. As medical students, we believe that it is our responsibility to be actively involved in fostering global collaboration to promote and implement medical education technology.

As medical students, it is our responsibility to be actively involved in fostering global collaboration to promote and implement medical education technology.

## SOLUTIONS FOR MAINTAINING ELEARNING

Effective hybrid teaching delivery models that combine in-person teaching with online pre-reading material are needed globally to maintain continuity of medical education. Implementing solutions that have the potential to mitigate issues with Internet speed and connectivity in LMICs include focusing on eLearning content that can be delivered and accessed asynchronously, such as lecture recordings and other downloadable content (e.g., videos of dissections, patient examinations, clinical skills, animations, podcasts, eModules, or transcripts).

Additionally, learning platform developers need to optimize their products for functioning on mobile devices and in data saver mode to ensure affordability and accessibility for students who do not own a personal computer. Teaching students self-directed learning skills, for which they may not have received training before, has the potential to increase knowledge retention even in the absence of in-person education.^9^

Utilizing social media to disseminate educational resources and to share best practices of adapting them to local conditions could allow for a bottom-up approach in which the medical students from HICs and LMICs could empower each other in overcoming adversities of the current situation.

Transferring existing eLearning platforms and resources from HICs to LMICs is another potential solution. Nevertheless, the software cannot be directly implemented without adjusting for language, cultural, and country-specific needs.^8^ As such, if TEL solutions are to be transferred from HICs to LMICs and successfully implemented, they need to be designed using principles of frugal and responsible innovation (e.g., capacity and needs assessment and consideration of local environmental factors).^13^

## ENCOURAGING MEDICAL STUDENTS' COLLABORATION TO IMPLEMENT BOTTOM-UP SOLUTIONS

Even though all these ideas have been suggested, their implementation proves to be challenging, and the problem has yet to be resolved. Due to the limited preparation, most of the effort to introduce TEL in LMICs has been top-down and did not involve students, who are arguably the most important stakeholders.^9^ We are convinced that medical students in both HICs and LMICs should actively seek opportunities to engage in the process of improving TEL. Successful implementation depends on collecting feedback on novel teaching methods, engaging in medical education research, and advocating for the establishment of international collaborations and reciprocal exchange programs.

Successful implementation of these solutions depends on collecting feedback from medical students who are arguably the most important stakeholders.

This notion can be supported by the recent emergence of local social media initiatives originating from the United Kingdom to have a global reach. Social media platforms, including Facebook, Instagram, Slack, WhatsApp, and especially Twitter, showed the effectiveness of students' and junior doctors' networks in the United Kingdom during the COVID-19 pandemic. Networks such as SMILE (Sustaining Medical education In a Lockdown Environment) Medical Education (@Lockdown_MedEd) or COVID Medical Education (@EdCovid) helped supplement teaching lost due to the pandemic and helped in developing much needed self-regulated learning skills with free webinars and peer tutorials. These networks have been created using a bottom-up approach and continuous feedback to tailor content to students' needs. The use of social media and online webinars allows for such resources to be disseminated and accessed globally, democratizing access to high-quality teaching (the SMILE Medical Education Facebook group has approximately 13,800 members as of early October 2020). We think that this bottom-up approach could be emulated on a global scale, as connecting students and junior doctors who have a passion for medical education has been shown to be a valuable complementary resource during the pandemic, largely dependent on using technology. Moreover, introducing technology into medical education creates an opportunity for students to develop collaborative skills, improve adaptability, and prepare them for future clinical practice, which will likely rely on technological literacy of doctors to a greater extent.^14^

Another way to catalyze international partnerships of students can be by following a model developed by research collaboratives such as GlobalSURG, a global academic network of practicing surgeons from around the world that conducts international research into surgical outcomes.^15^ GlobalSURG is open not only to doctors, but also nurses, medical students, and researchers, allowing them to participate in multicenter, prospective cohort studies regardless of their previous experience and expertise.^16^^,^^17^ The current network includes over 5,000 collaborators from more than 100 countries, creating an inclusive environment. To ensure easy delivery in diverse local conditions, each study is designed so that it does not require additional resources or funding. Moreover, GlobalSURG offers their study documents translated into several languages to facilitate local dissemination and adoption. By creating an inclusive environment, such initiatives empower grass-root academics and build research capacity locally.

We propose a similar framework to foster cooperation for improving eLearning delivery in LMICs by creating opportunities for exchanging study materials, discussing ideas on delivery methods, and sharing best practices. However, this new platform, while driven by medical students, should also bring together academics, teaching fellows, and lecturers. By exchanging experiences and sharing knowledge of locally available infrastructure, collaborators could brainstorm ideas and tailor the eLearning content to the needs of LMIC institutions. Results of these discussions should be summarized and published, making them available to the academic community worldwide. Outcomes of this cooperation could also apply to other fields and serve as guidelines for creating online resources. For example, Abidi et al.^18^ drew from their experience in Pakistan and described a roadmap for offering a massive open online course at an LMIC institution. Similar articles would have a crucial role in accelerating the creation of widely available eLearning platforms and online educational content. Moreover, a transfer of technological know-how, both between institutions and students, would help with efficient administration of learning environments and maximize their usefulness.

We propose an international networking framework to foster cooperation for improving eLearning delivery in LMICs by creating opportunities for exchanging study materials, ideas, and best practices.

Disruption of medical education globally, caused by the burden of COVID-19, is likely to continue through the coming months or even years. TEL is going to play a crucial role as a supplement to conventional learning methods in preparing medical students for the challenges of our profession. Tailored hybrid teaching methods must be developed and implemented to prevent further broadening of the gap in access to high-quality education between HICs and LMICs. Medical students of our generation across the world have an instrumental role to play. It is our responsibility to cooperate with university faculty members to drive changes to get the education necessary to provide the highest standard of care for our future patients globally during the COVID-19 pandemic and in the future.

## Notes

**Peer Reviewed**

**First published online:** January 14, 2021.
